# Supplementary material for: Development of an Eco-Friendly Nanogel Incorporating Pectis brevipedunculata Essential Oil as a Larvicidal Agent Against Aedes aegypti
Source: Pharmaceutics. 2024 Oct 18;16(10):1337. doi: 10.3390/pharmaceutics16101337 (PMC11510620; doi:10.3390/pharmaceutics16101337)
Supplement: Supplementary file 1 [file pharmaceutics-16-01337-s001.zip › pharmaceutics-3249322-SI.pdf]

SUPPORTING INFORMATION

Development of an Eco-Friendly Nanogel Incorporating *Pectis brevipedunculata* Essential Oil as Larvicidal Agent Against *Aedes aegypti*

Estela Mesquita Marques[1], Raiene Lisboa Rocha[1], Clenilma Marques Brandão[2], Júlia karla Albuquerque Melo Xavier[1], Marcos Bispo Pinheiro Camara[1], Caritas de Jesus Silva Mendonça[3], Roberto Batista de Lima[4], Melissa Pires Souza[6], Emmanoel Vilaça Costa[5,6], and Renato Sonchini Gonçalves[1]*

1. Laboratory of Chemistry of Natural Products, Department of Chemistry, Federal University of Maranhão (UFMA), São Luís 65080-805, Brazil;
2. Department of Chemistry, Federal Institute of Maranhão (IFMA), São Luis 65075-441, Brazil;
3. Center for Fuels, Catalysis, and Environment (NCCA), Department of Chemistry, Federal University of Maranhão (UFMA), São Luís 65080-805, Brazil;
4. Department of Chemistry, Federal University of Maranhão, São Luís 65080-805, Brazil;
5. Department of Chemistry, Federal University of Amazonas (UFAM), Manaus 69080-900, AM, Brazil.
6. Postgraduate Program in Chemistry, Federal University of Amazonas (UFAM), Manaus 69080-900, AM, Brazil.

* Correspondence: renato.sg@ufma.br; Tel.: +55-98-985149235

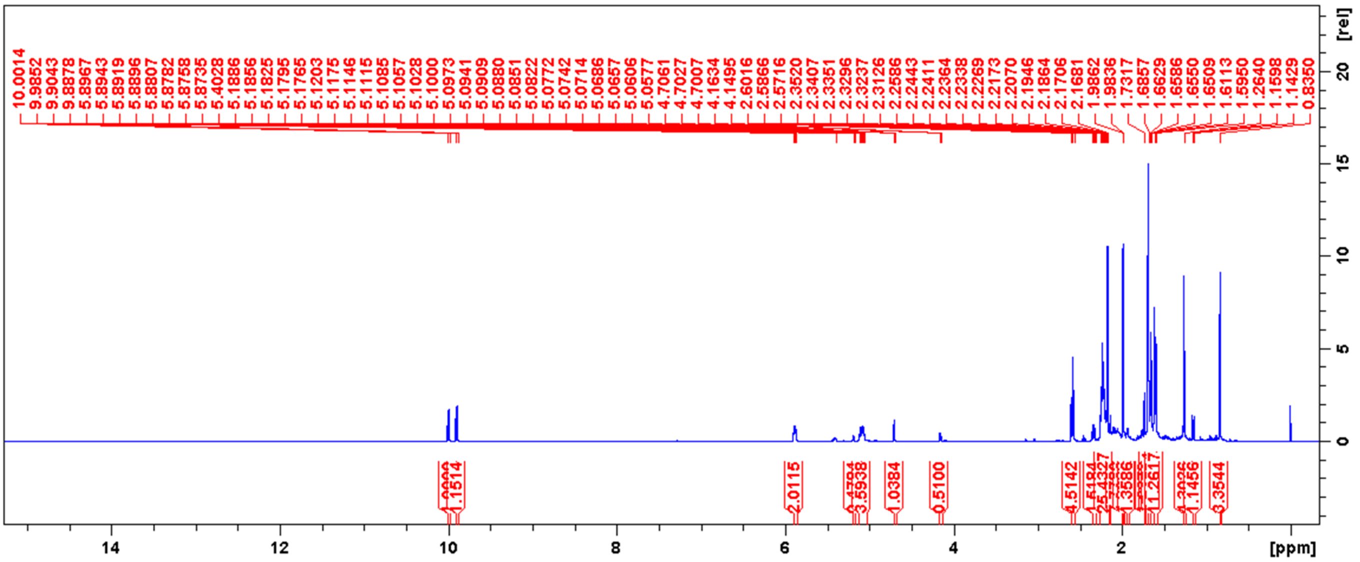

Figure S1. ^{1}H NMR spectra of EO*Pb*

(A)

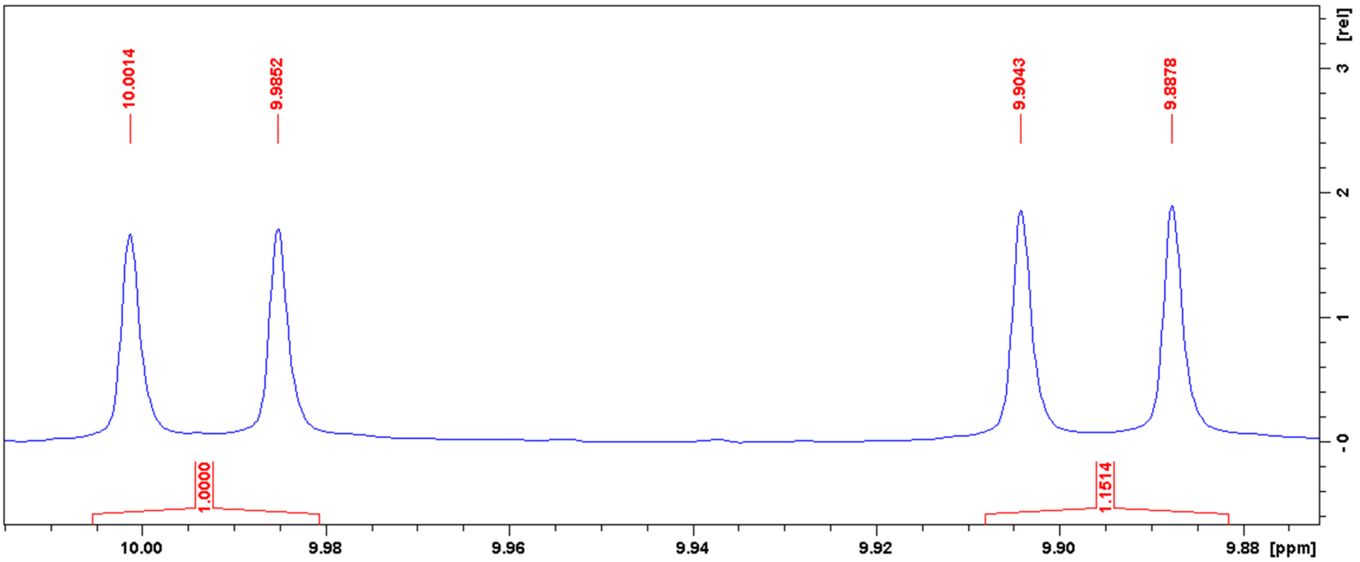

(B)

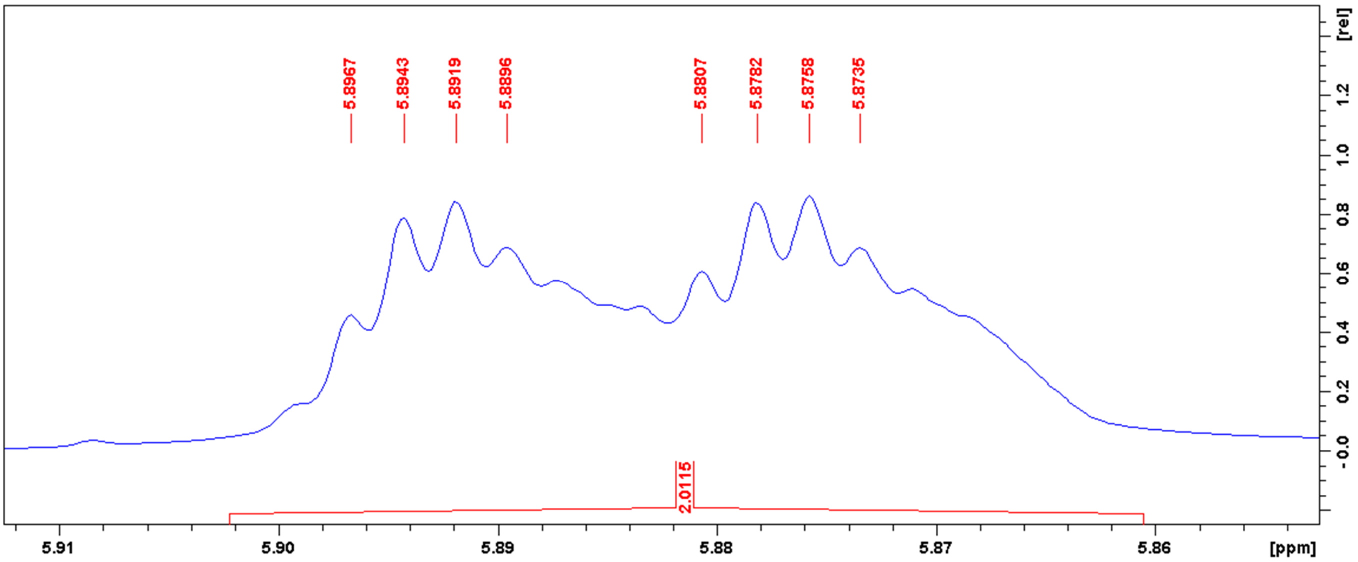
5.8967
5.8943
5.8919
5.8896
5.8807
5.8782
5.8758
5.8735
2.0115
5.91
5.90
5.89
5.88
5.87
5.86
[ppm]
[rel]
-0.0
0.2
0.4
0.6
0.8
1.0
1.2

(C)

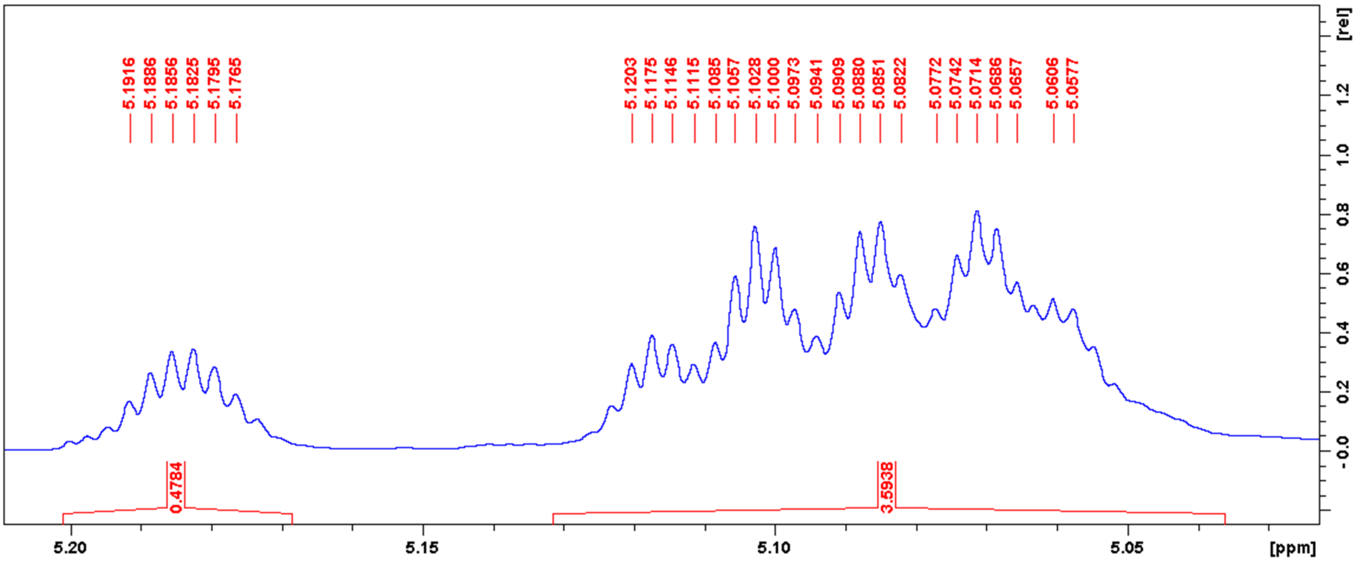
5.1916
5.1886
5.1856
5.1825
5.1795
5.1765
5.1203
5.1175
5.1146
5.1115
5.1085
5.1057
5.1028
5.1000
5.0973
5.0941
5.0909
5.0880
5.0851
5.0822
5.0772
5.0742
5.0714
5.0686
5.0657
5.0606
5.0577
0.4784
3.5938
5.20
5.15
5.10
5.05
[ppm]
[rel]
-0.0
0.2
0.4
0.6
0.8
1.0
1.2

(D)

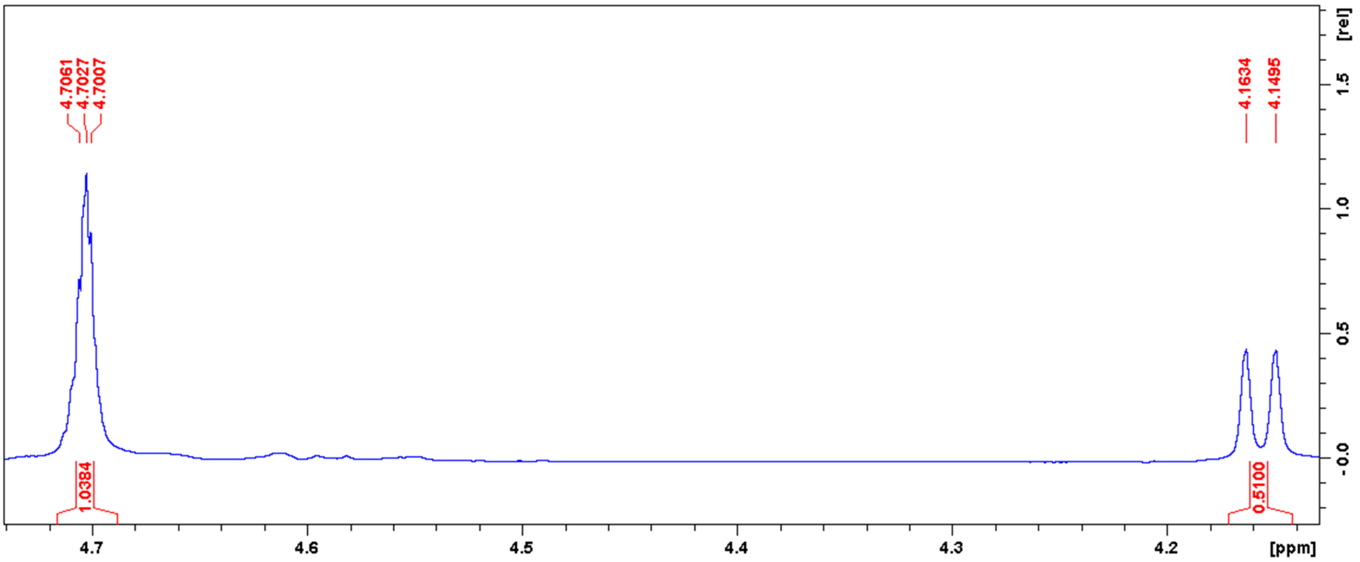
4.7061
4.7027
4.7007
4.1634
4.1495
1.0384
0.5100
[rel]
1.5
1.0
0.5
-0.0
4.7
4.6
4.5
4.4
4.3
4.2
[ppm]

(E)

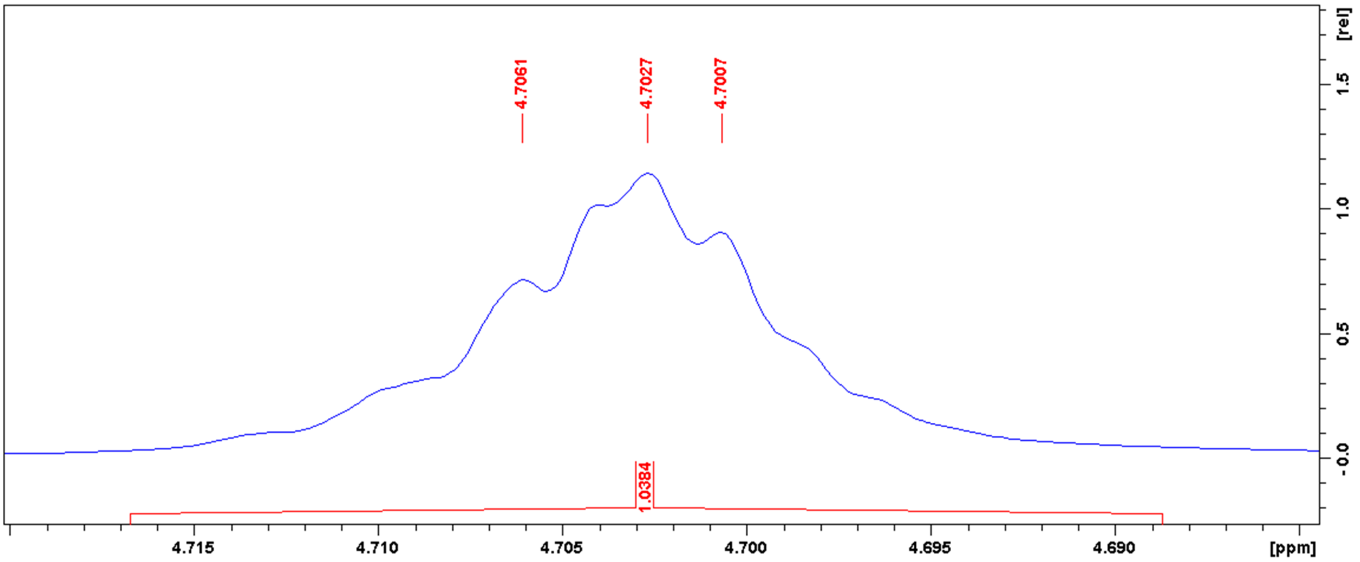
4.7061
4.7027
4.7007
1.0384
[rel]
1.5
1.0
0.5
-0.0
4.715
4.710
4.705
4.700
4.695
4.690
[ppm]

(F)

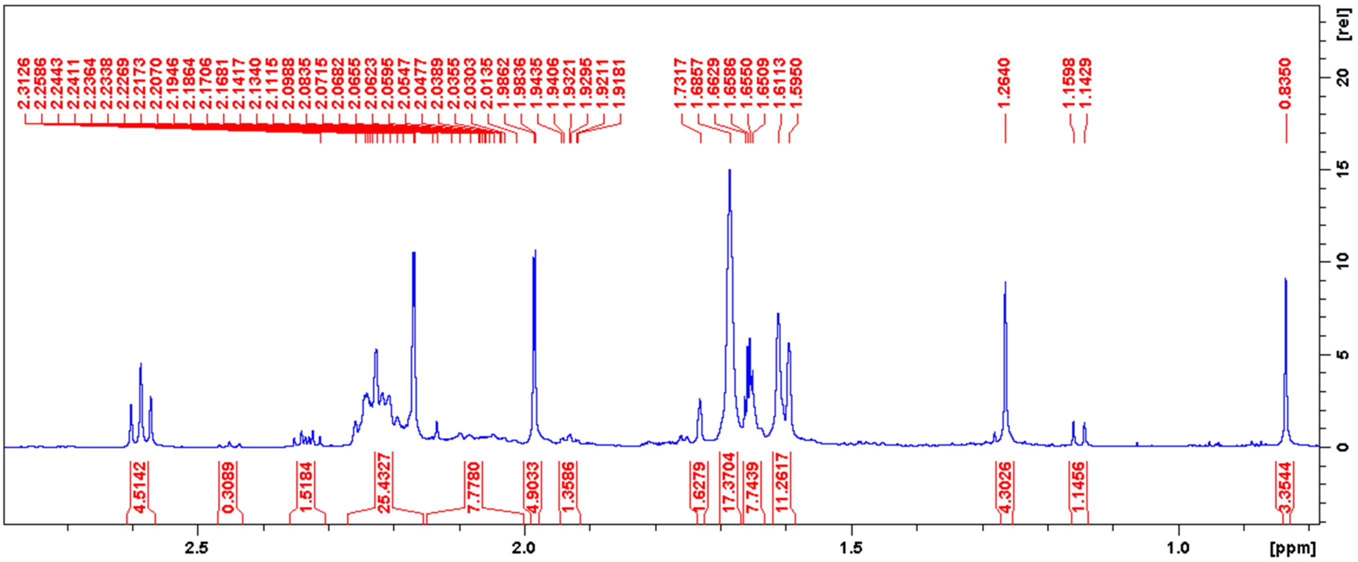
2.3126 2.2586 2.2443 2.2411 2.2364 2.2338 2.2269 2.2173 2.2070 2.1946 2.1864 2.1706 2.1681 2.1417 2.1340 2.1115 2.0988 2.0835 2.0715 2.0682 2.0655 2.0623 2.0595 2.0547 2.0477 2.0389 2.0355 2.0303 2.0135 1.9862 1.9836 1.9435 1.9406 1.9321 1.9295 1.9211 1.9181
1.7317 1.6857 1.6629 1.6586 1.6550 1.6509 1.6113 1.5950
1.2640
1.1598 1.1429
0.8350
4.5142 0.3089 1.5184 25.4327 7.7780 4.9033 1.3586 1.6279 17.3704 7.7439 11.2617 4.3026 1.1456 3.3544
[rel]
[ppm]

(G)

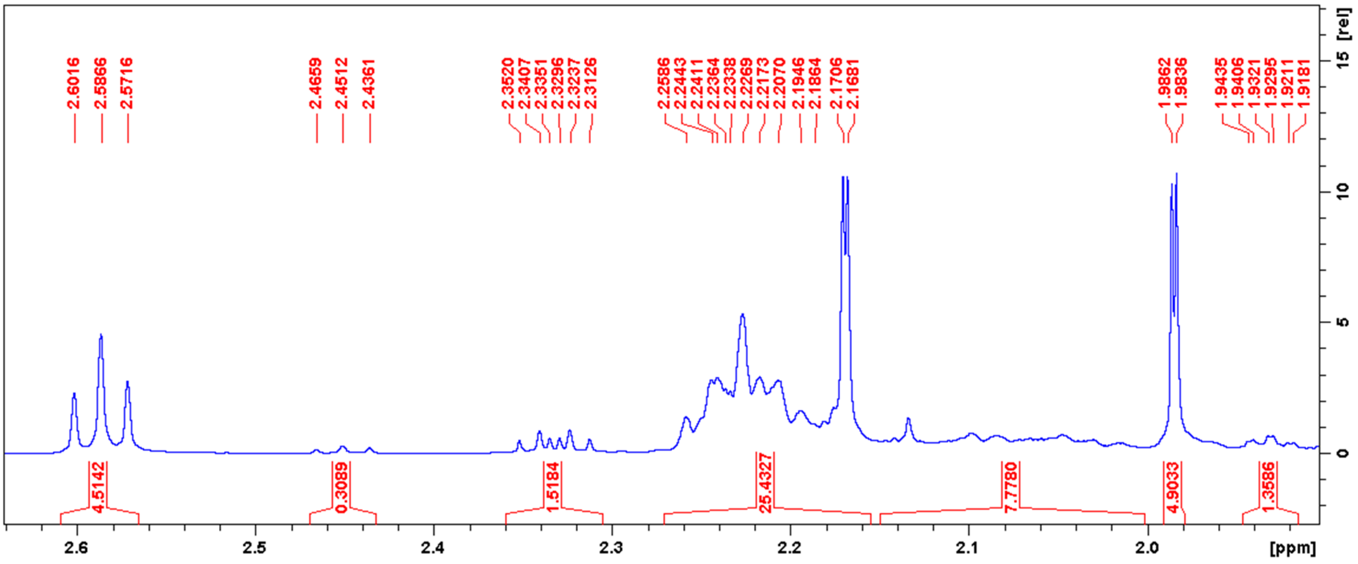
2.6016 2.5866 2.5716
2.4659 2.4512 2.4361
2.3520 2.3407 2.3351 2.3296 2.3237 2.3126
2.2586 2.2443 2.2411 2.2364 2.2338 2.2269 2.2173 2.2070 2.1946 2.1864 2.1706 2.1681
1.9862 1.9836
1.9435 1.9406 1.9321 1.9295 1.9211 1.9181
4.5142 0.3089 1.5184 25.4327 7.7780 4.9033 1.3586
[rel]
[ppm]

(H)

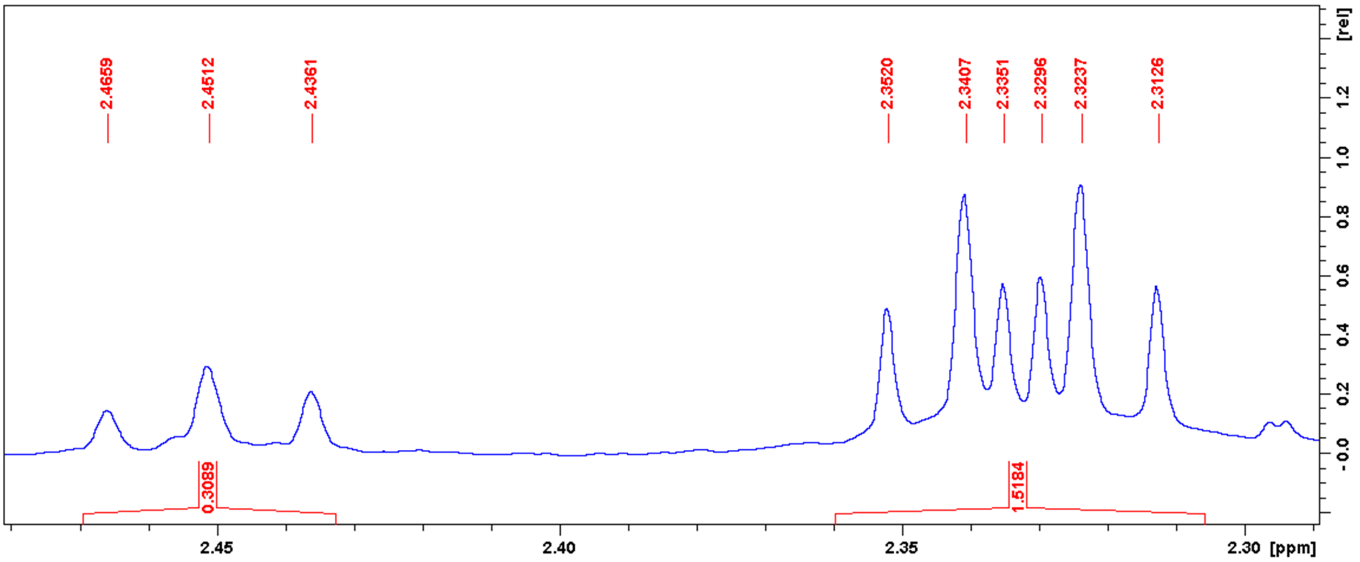

2.4659
2.4512
2.4361
2.3520
2.3407
2.3351
2.3296
2.3237
2.3126
0.3089
1.5184
2.45
2.40
2.35
2.30 [ppm]
[rel]

(I)

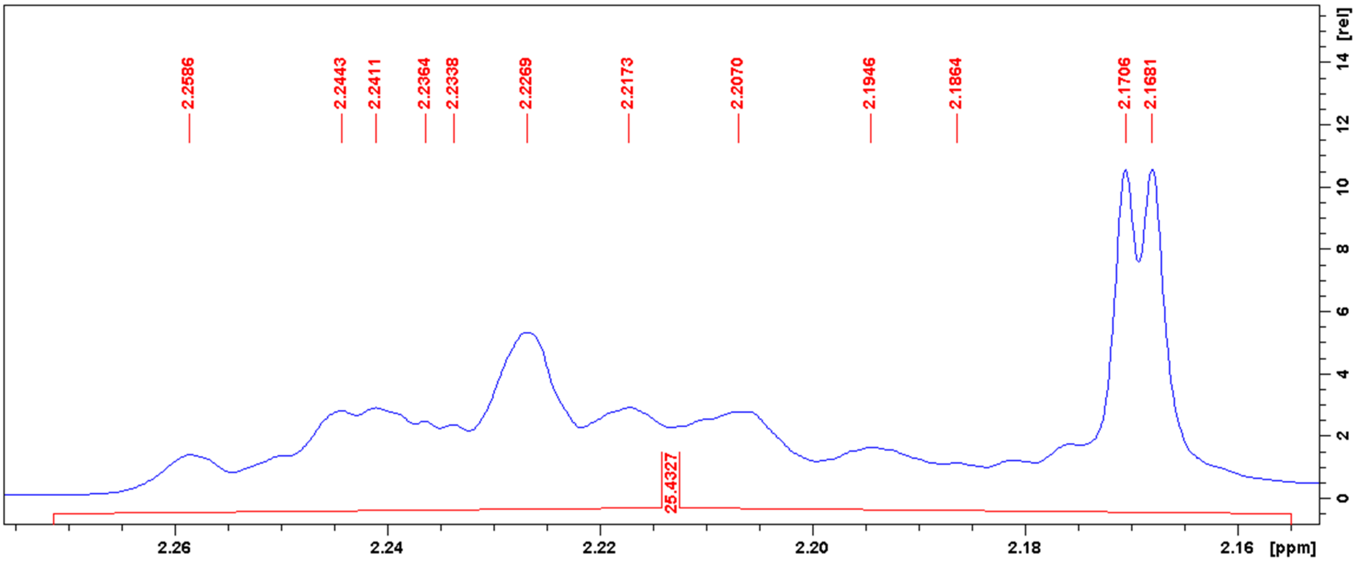

2.2586
2.2443
2.2411
2.2364
2.2338
2.2269
2.2173
2.2070
2.1946
2.1864
2.1706
2.1681
25.4327
2.26
2.24
2.22
2.20
2.18
2.16 [ppm]
[rel]

(J)

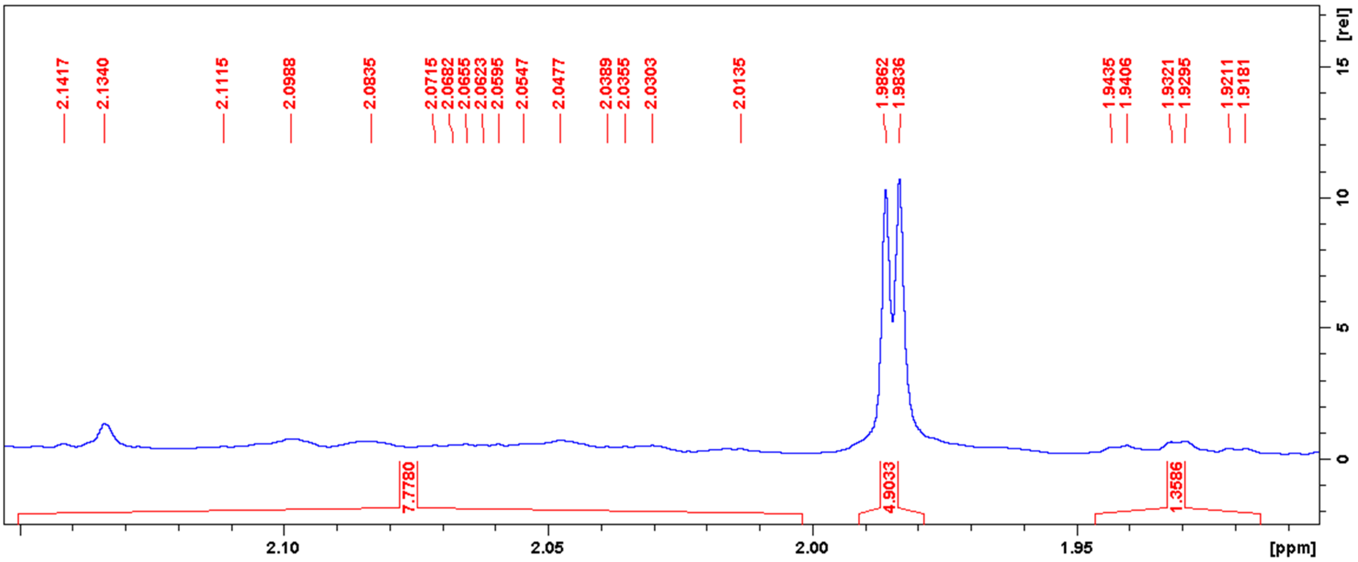
2.1417
2.1340
2.1115
2.0988
2.0835
2.0715
2.0682
2.0655
2.0623
2.0595
2.0547
2.0477
2.0389
2.0355
2.0303
2.0135
1.9862
1.9836
1.9435
1.9406
1.9321
1.9295
1.9211
1.9181
7.7780
4.9033
1.3586
[rel]
15
10
5
0
2.10
2.05
2.00
1.95
[ppm]

(K)

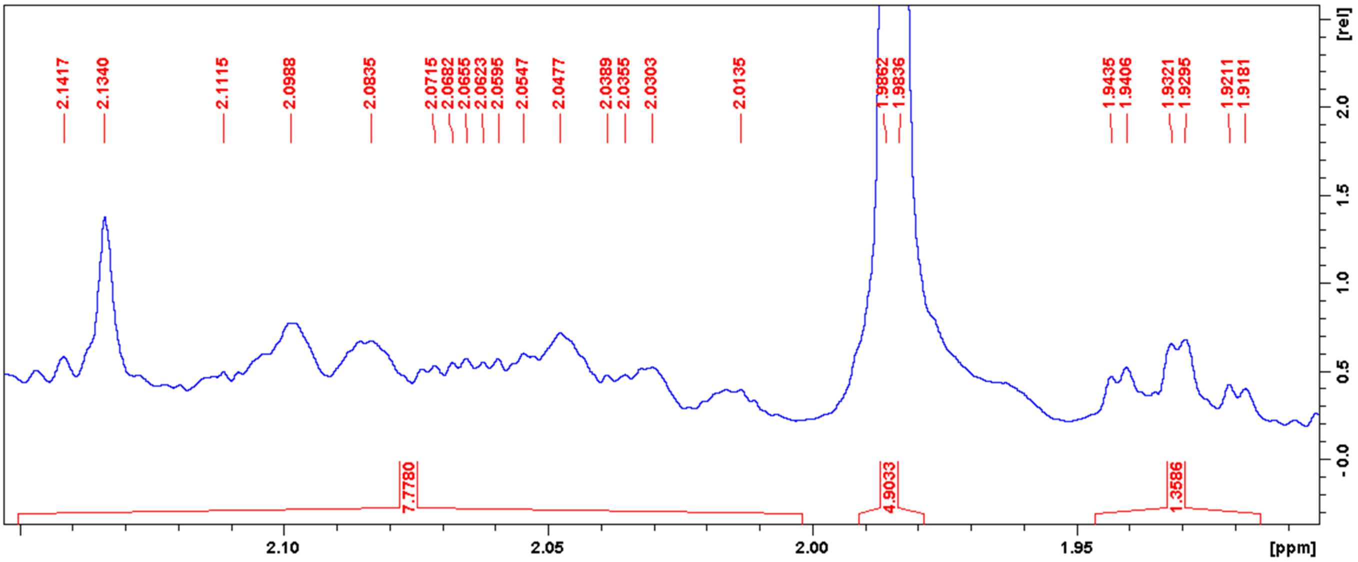
2.1417
2.1340
2.1115
2.0988
2.0835
2.0715
2.0682
2.0655
2.0623
2.0595
2.0547
2.0477
2.0389
2.0355
2.0303
2.0135
1.9862
1.9836
1.9435
1.9406
1.9321
1.9295
1.9211
1.9181
7.7780
4.9033
1.3586
[rel]
2.0
1.5
1.0
0.5
-0.0
2.10
2.05
2.00
1.95
[ppm]

(L)

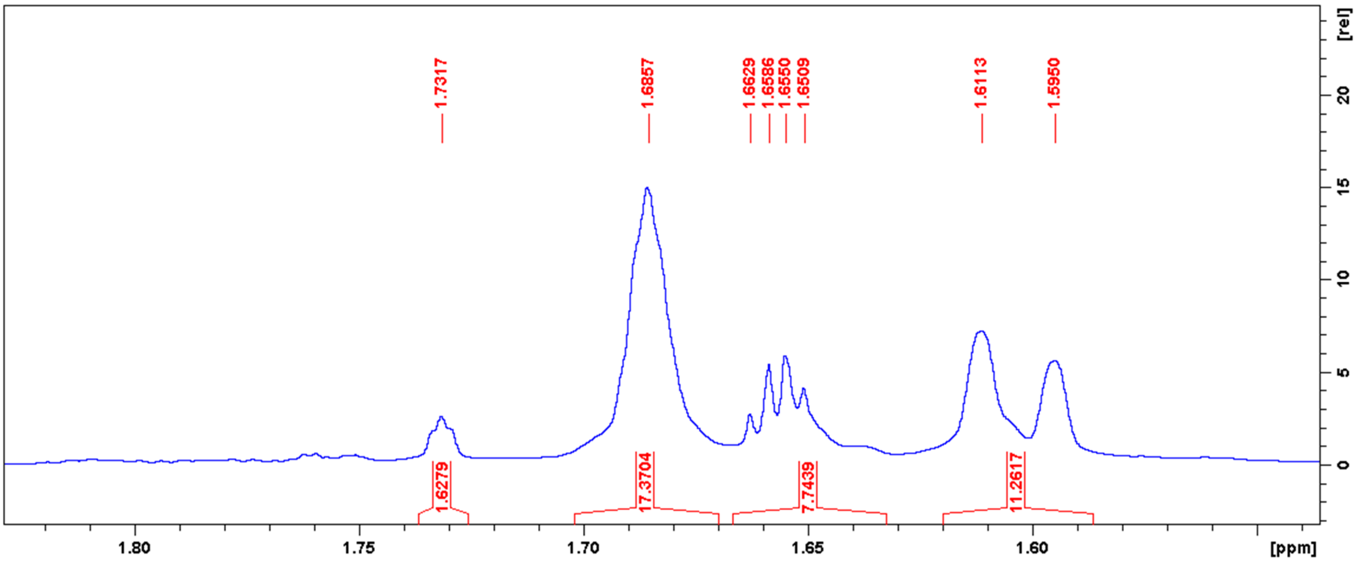

(M)

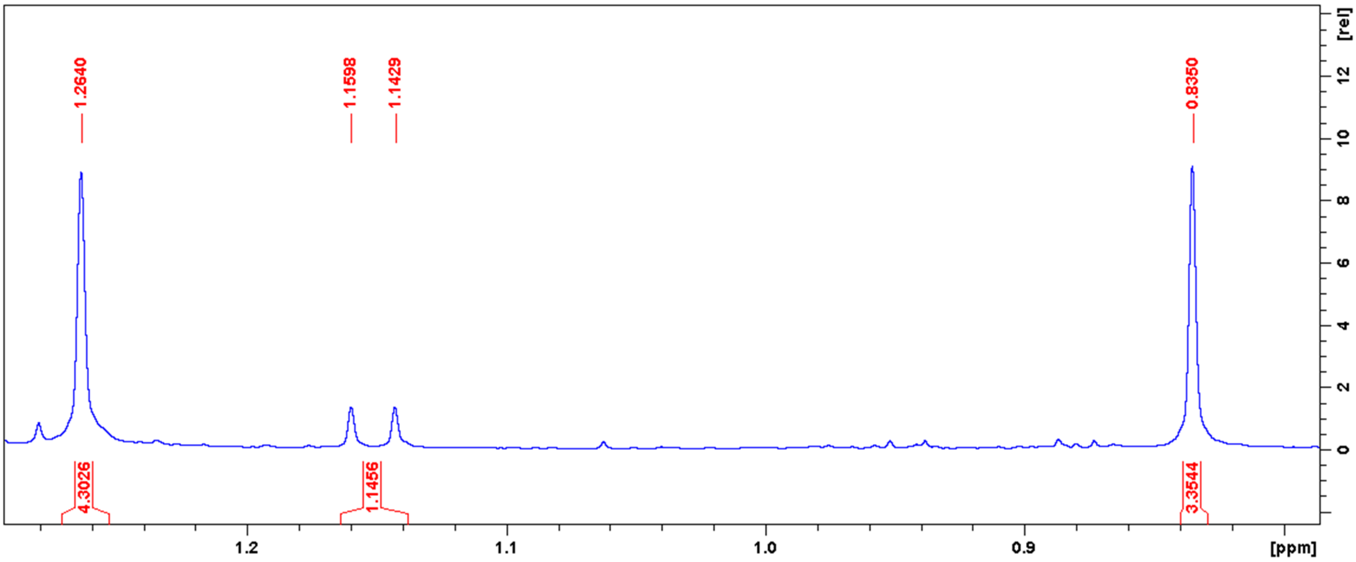

Figure S2. Expansion of ^{1}H NMR spectrum of EO*Pb* (A) to (M)

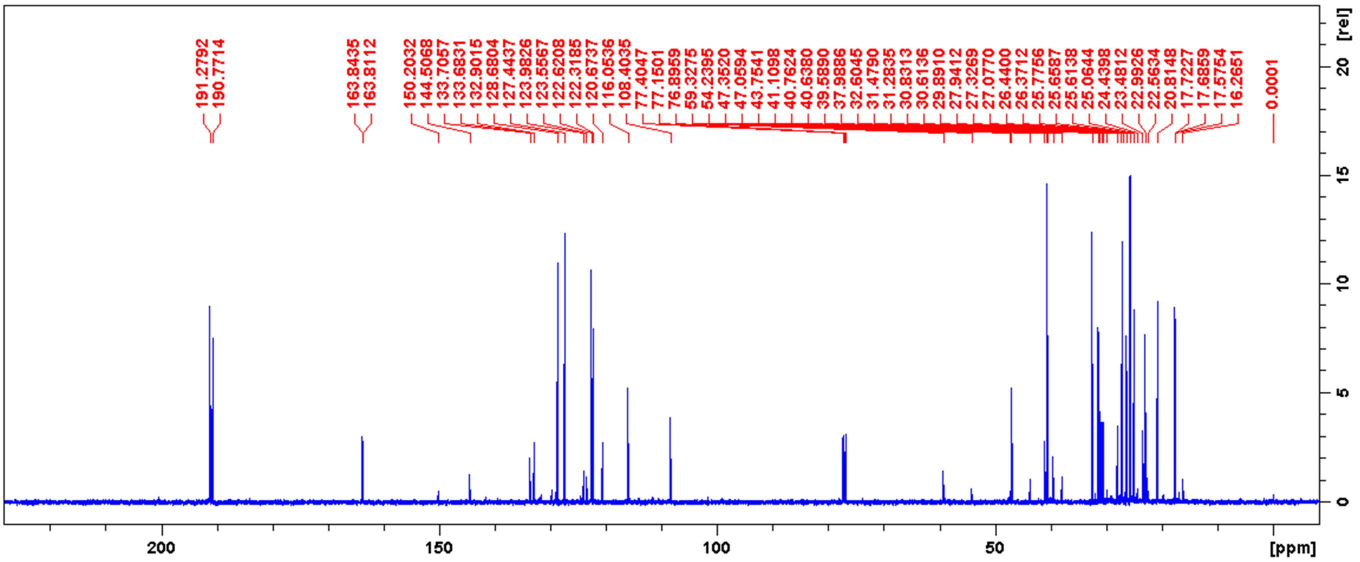

Figure S3. ^{13}C NMR spectra of EO*Pb*

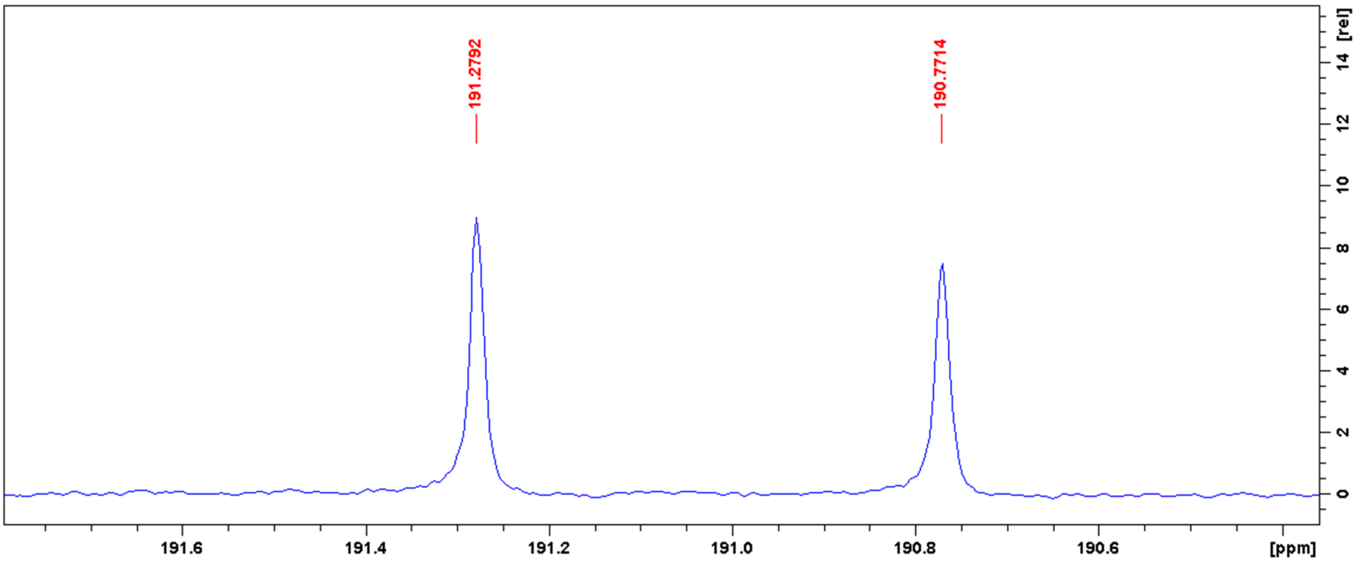

(A)

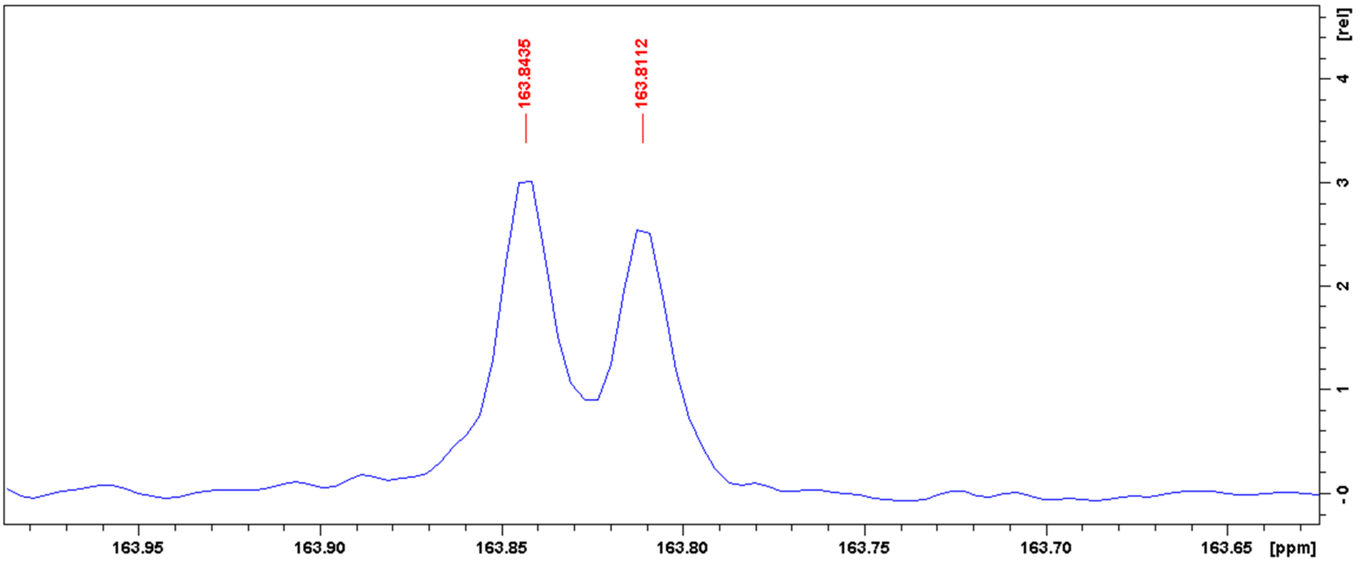
163.8435
163.8112
[rel]
4
3
2
1
0
163.95
163.90
163.85
163.80
163.75
163.70
163.65
[ppm]

(B)

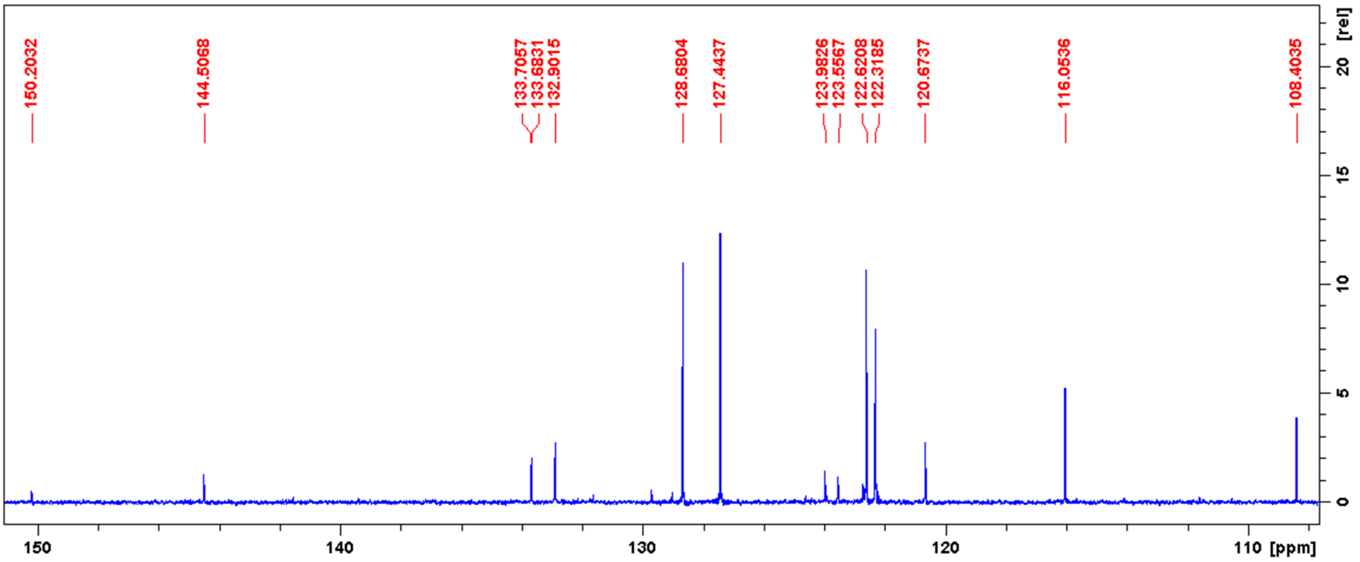
150.2032
144.5068
133.7057
133.6831
132.9015
128.6804
127.4437
123.9826
123.5567
122.6208
122.3185
120.6737
116.0536
108.4035
[rel]
20
15
10
5
0
150
140
130
120
110
[ppm]

(C)

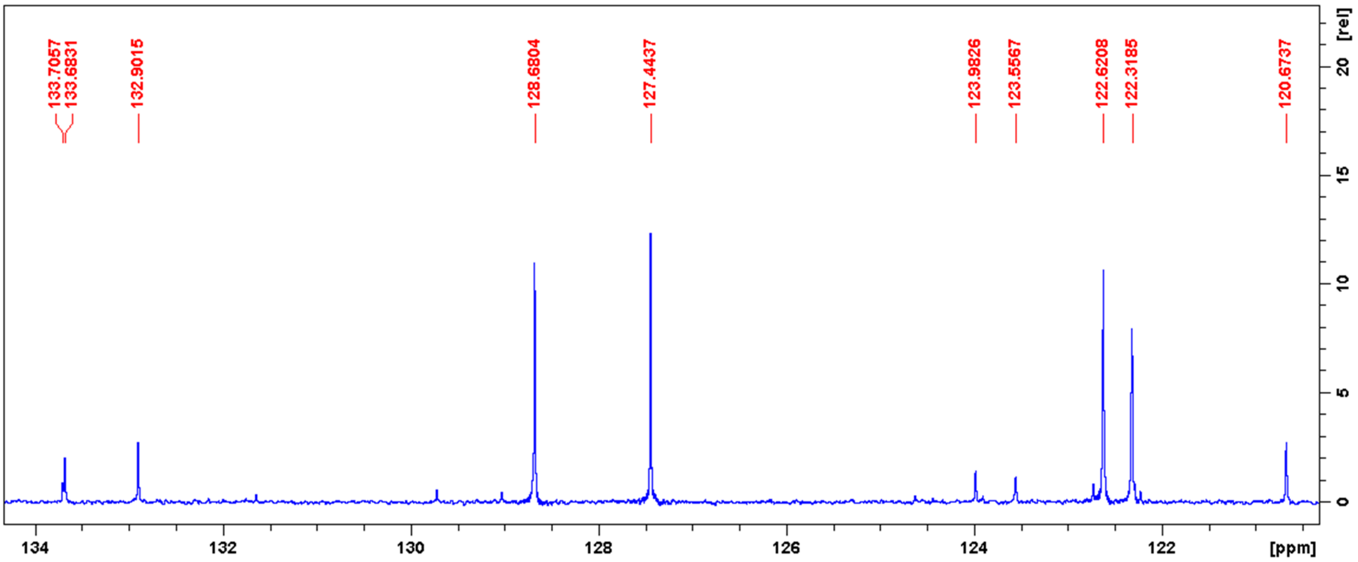
133.7057
133.6831
132.9015
128.6804
127.4437
123.9826
123.5567
122.6208
122.3185
120.6737
[rel]
20
15
10
5
0
134
132
130
128
126
124
122
[ppm]

(D)

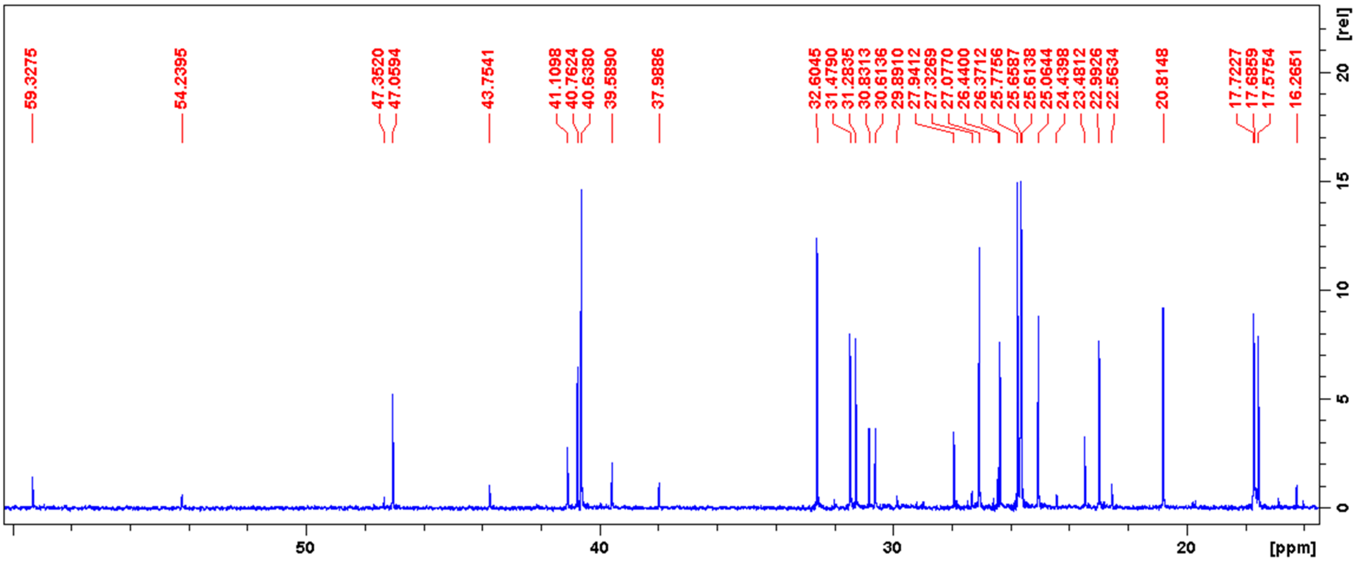
59.3275
54.2395
47.3520
47.0594
43.7541
41.1098
40.7624
40.6380
39.5890
37.9886
32.6045
31.4790
31.2835
30.8313
30.6136
29.8910
27.9412
27.3269
27.0770
26.4400
26.3712
25.7756
25.6587
25.6138
25.0644
24.4398
23.4812
22.9926
22.5634
20.8148
17.7227
17.6859
17.5754
16.2651
[rel]
20
15
10
5
0
50
40
30
20
[ppm]

(E)

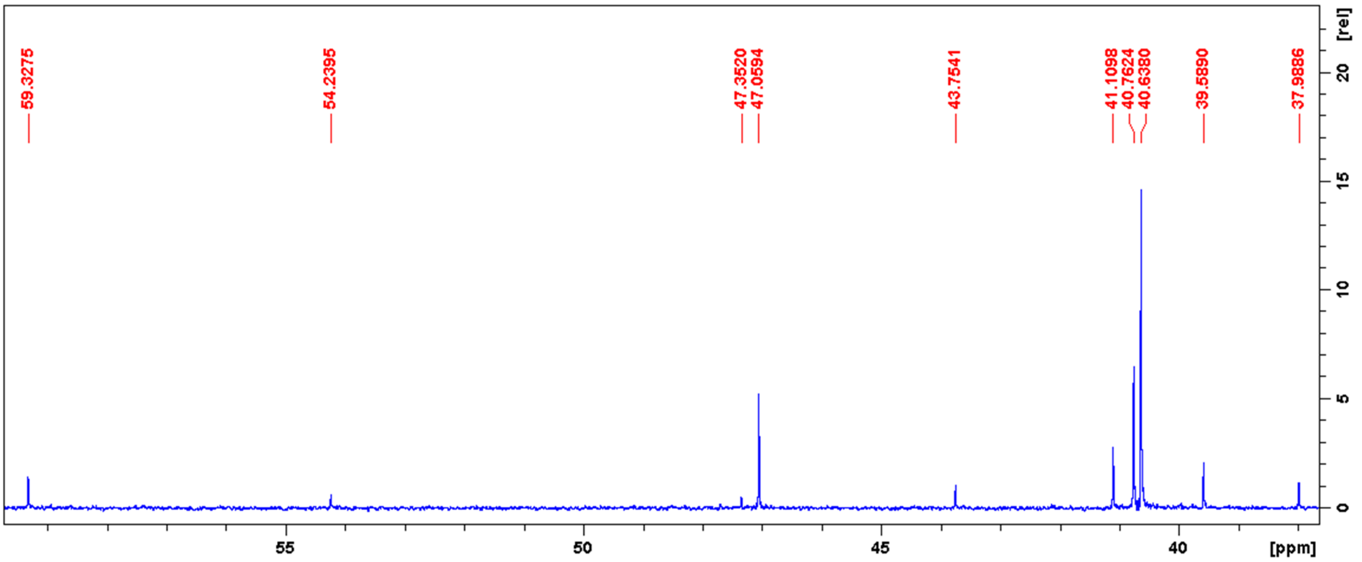
59.3275
54.2395
47.3520
47.0594
43.7541
41.1098
40.7624
40.6380
39.5890
37.9886
[rel]
20
15
10
5
0
55
50
45
40
[ppm]

(F)

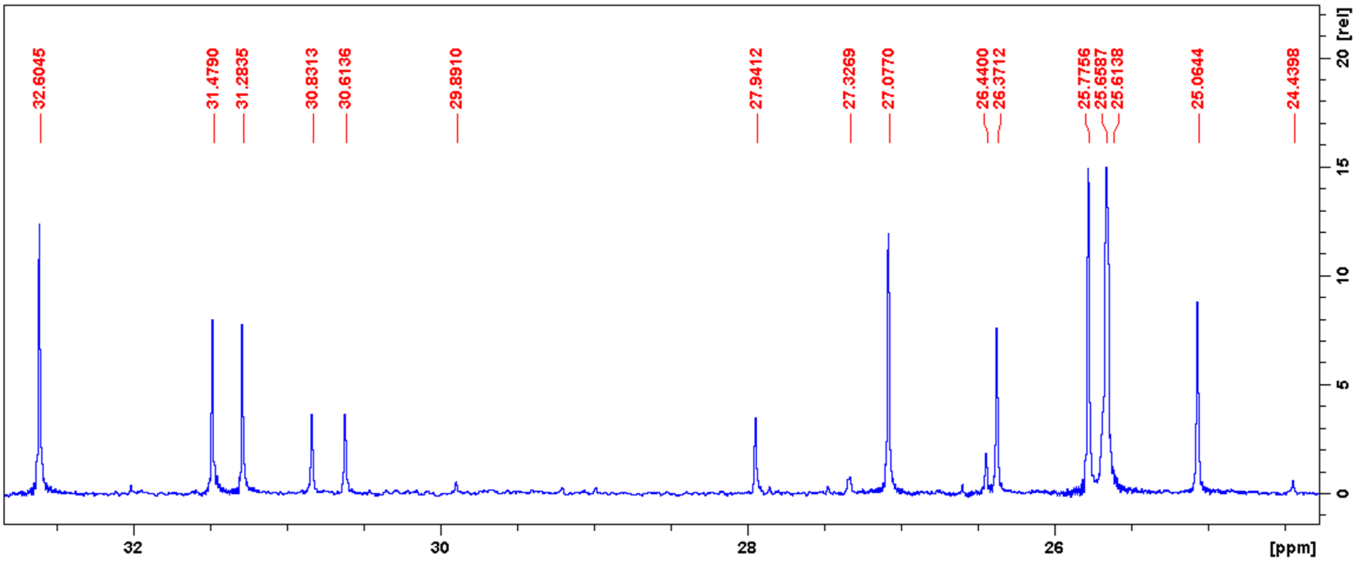
32.6045
31.4790
31.2835
30.8313
30.6136
29.8910
27.9412
27.3269
27.0770
26.4400
26.3712
25.7756
25.6587
25.6138
25.0644
24.4398
[rel]
20
15
10
5
0
32
30
28
26
[ppm]

(G)

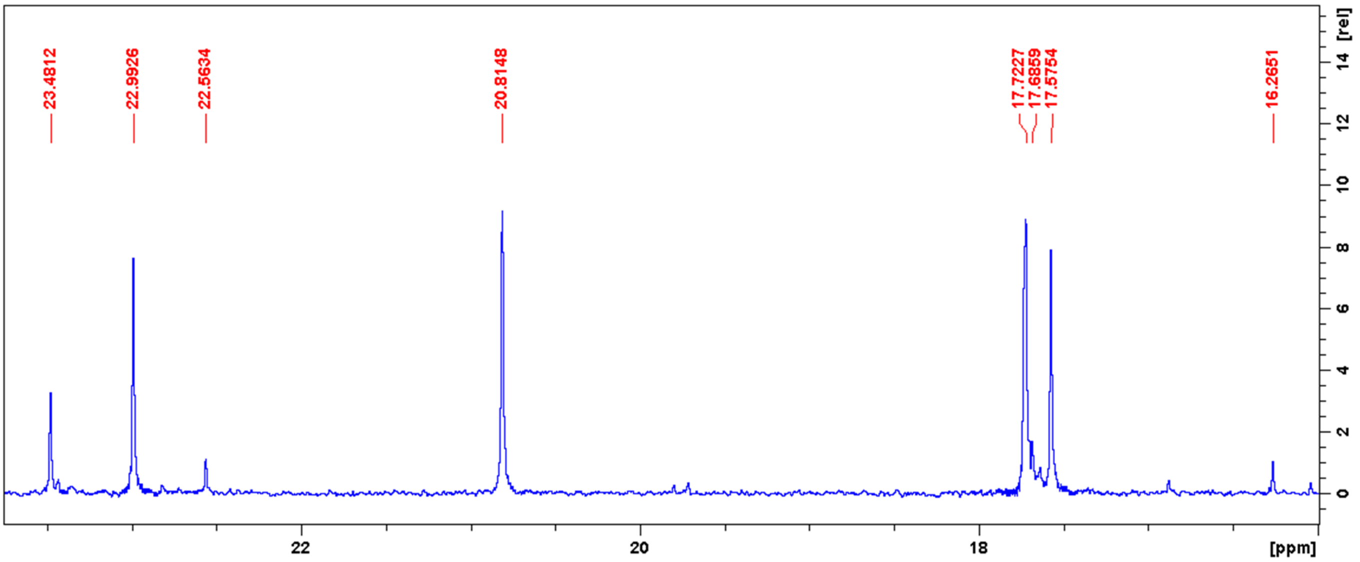

Figure S4. Expansion of ^{13}C NMR spectrum of EO*Pb* (A) to (G)

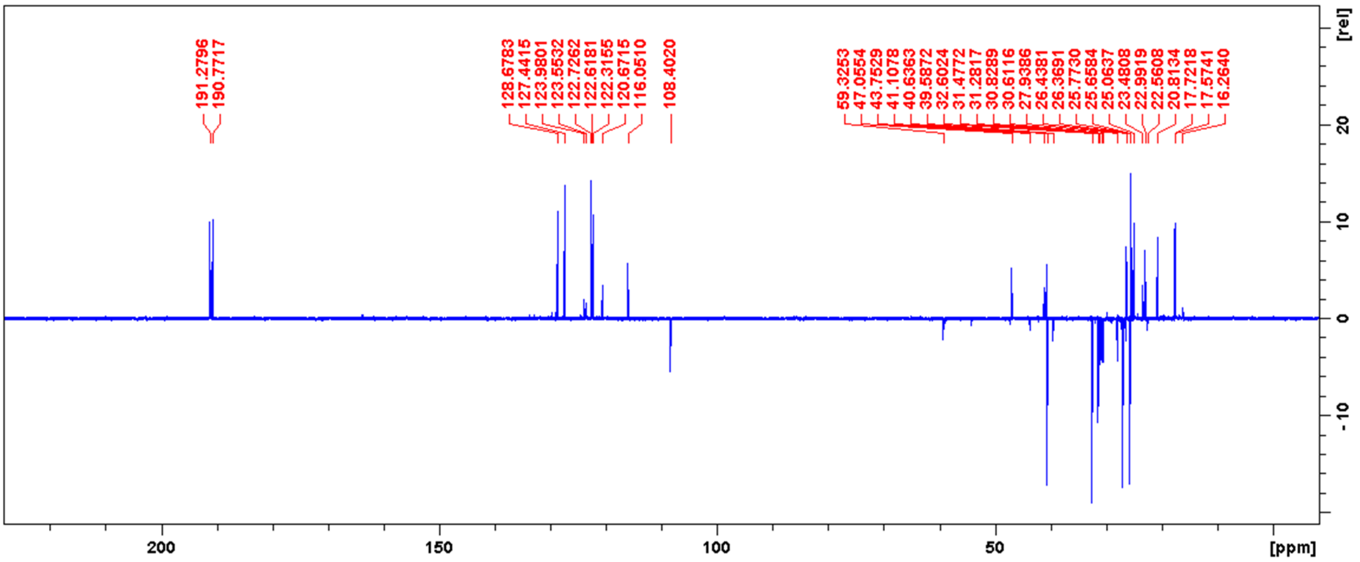

Figure S5. DEPT ^{13}C NMR spectrum of EO*Pb*

(A)

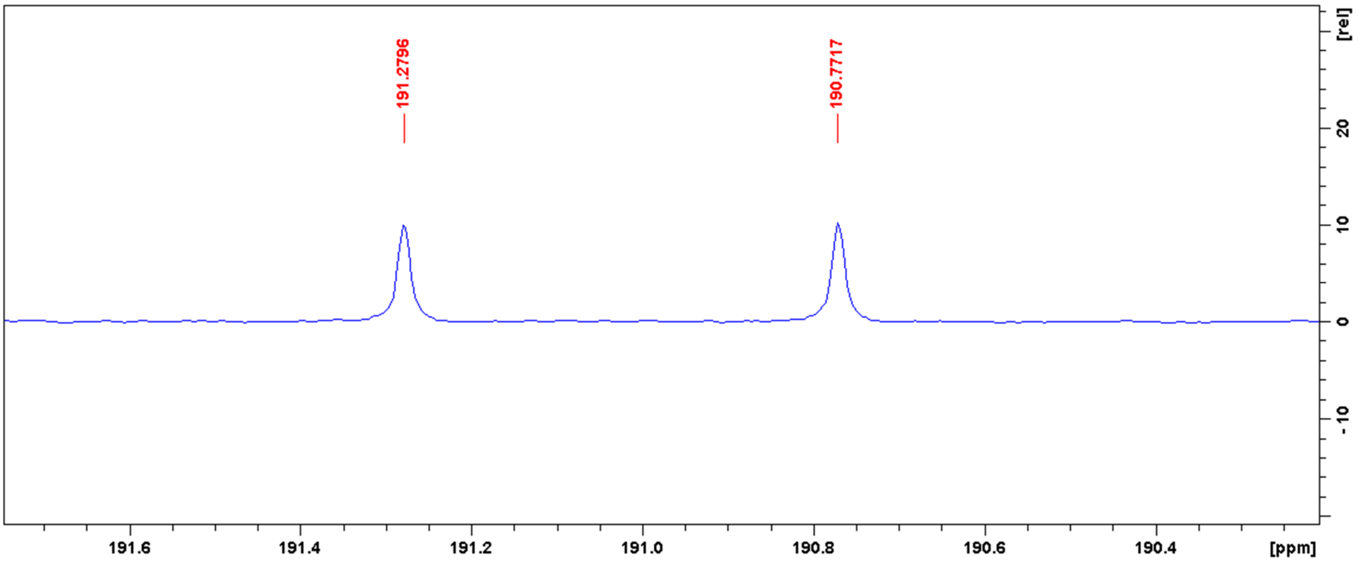
191.2796
190.7717
[rel]
20
10
0
-10
191.6
191.4
191.2
191.0
190.8
190.6
190.4
[ppm]

(B)

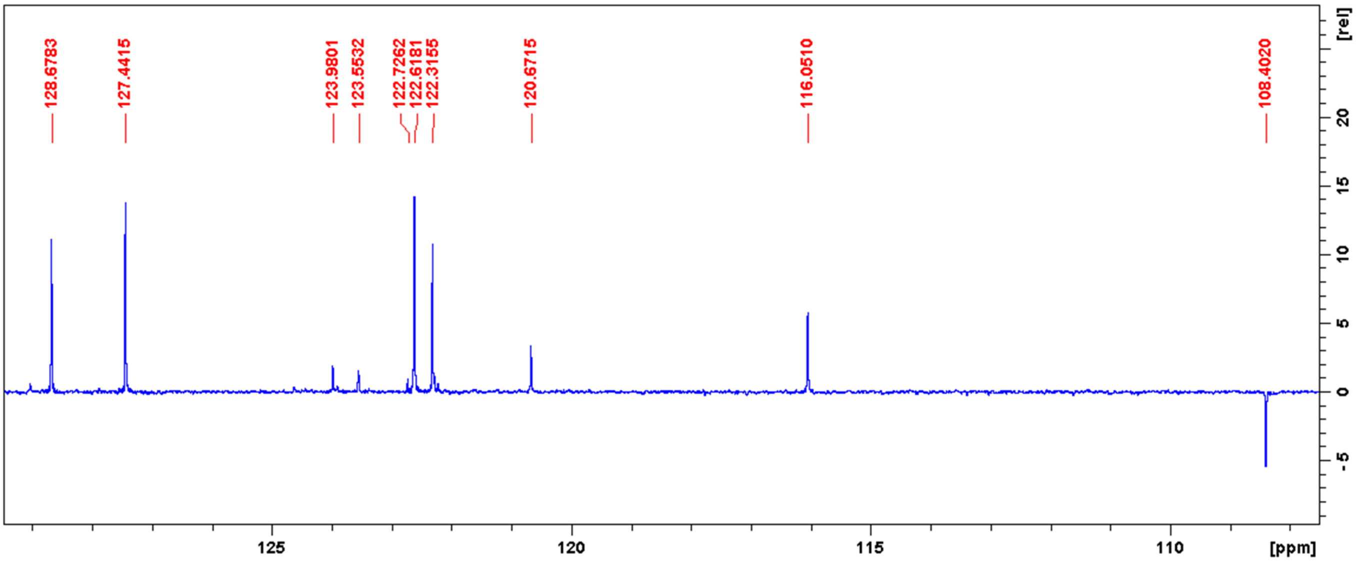
128.6783
127.4415
123.9801
123.5532
122.7262
122.6181
122.3155
120.6715
116.0510
108.4020
[rel]
20
15
10
5
0
-5
125
120
115
110
[ppm]

(C)

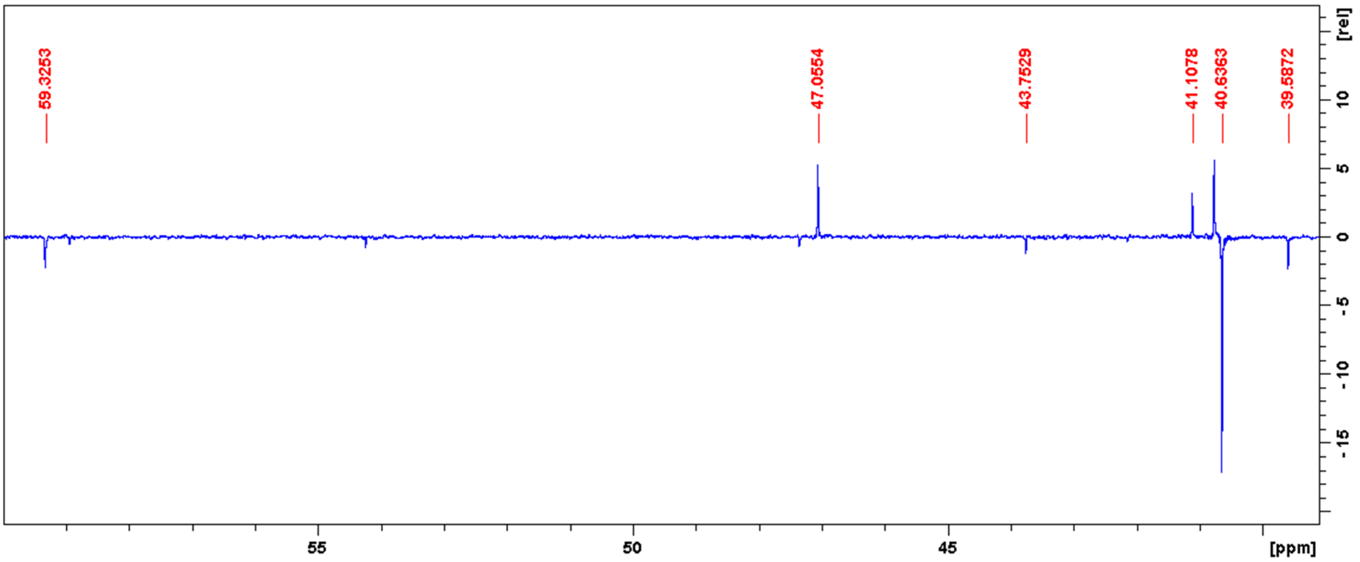
59.3253
47.0554
43.7529
41.1078
40.6363
39.5872
[rel]
55
50
45
[ppm]

(D)

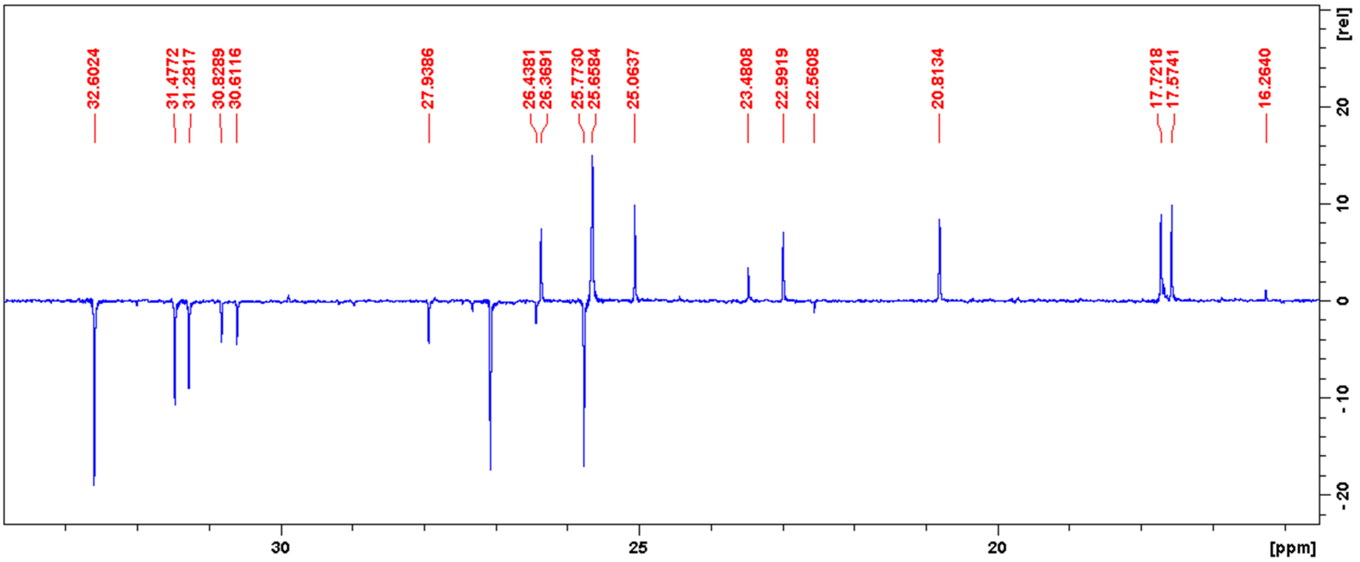
32.6024
31.4772
31.2817
30.8289
30.6116
27.9386
26.4381
26.3691
25.7730
25.6584
25.0637
23.4808
22.9919
22.5608
20.8134
17.7218
17.5741
16.2640
[rel]
30
25
20
[ppm]

(E)

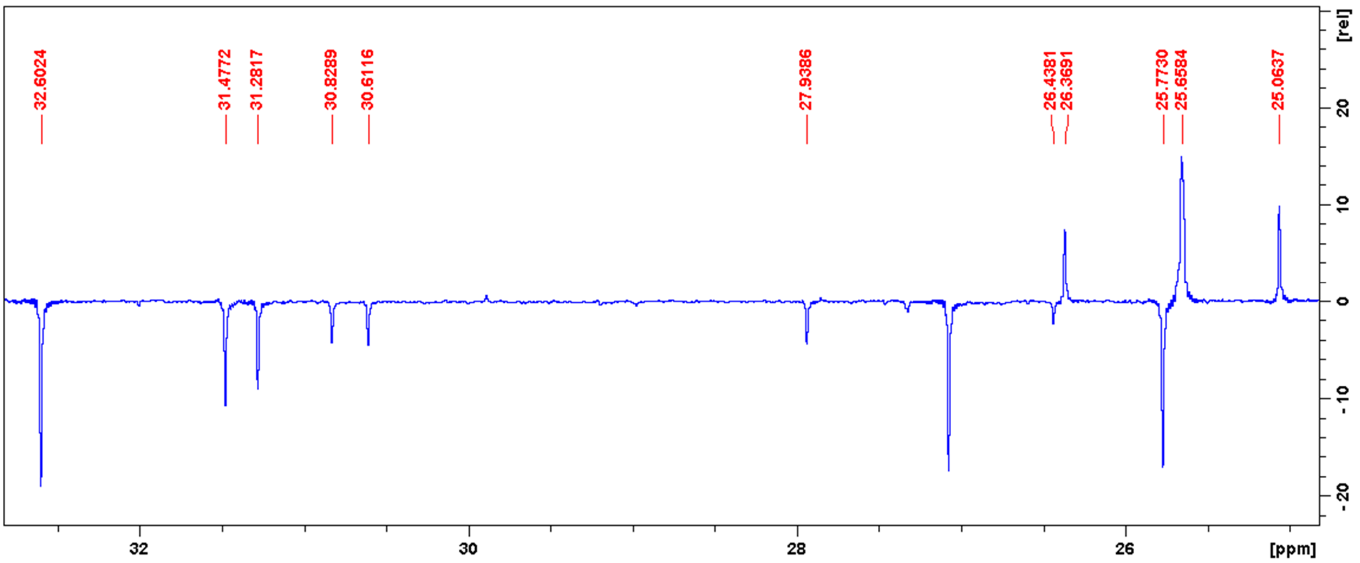

Figure S6. Expansion of DEPT ^{13}C NMR spectrum of EO*Pb* (A) to (E)

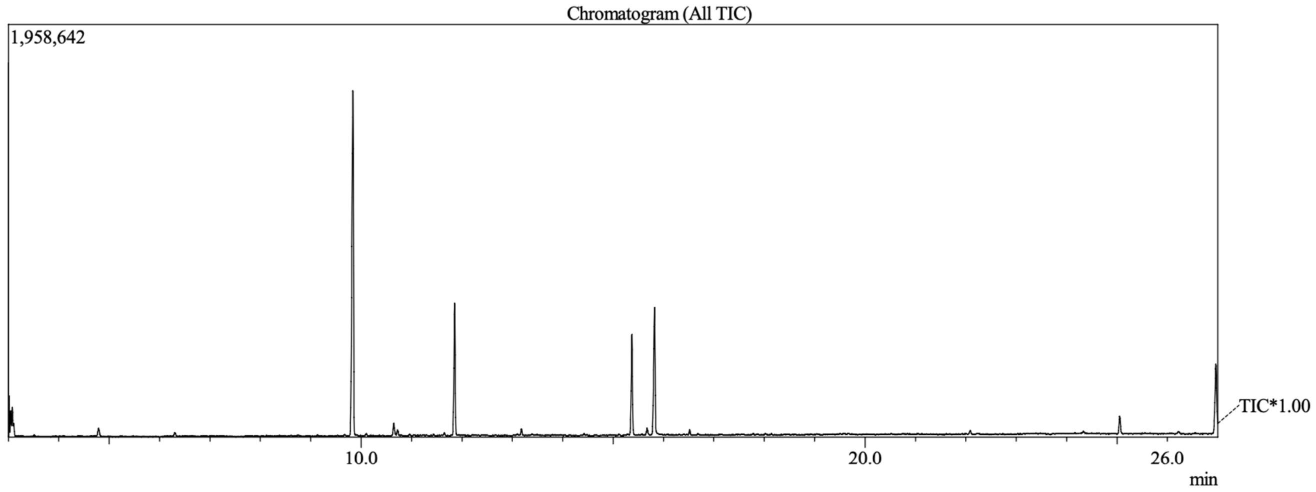

Figure S7. GC chromatogram of the nGF2002*Pb*

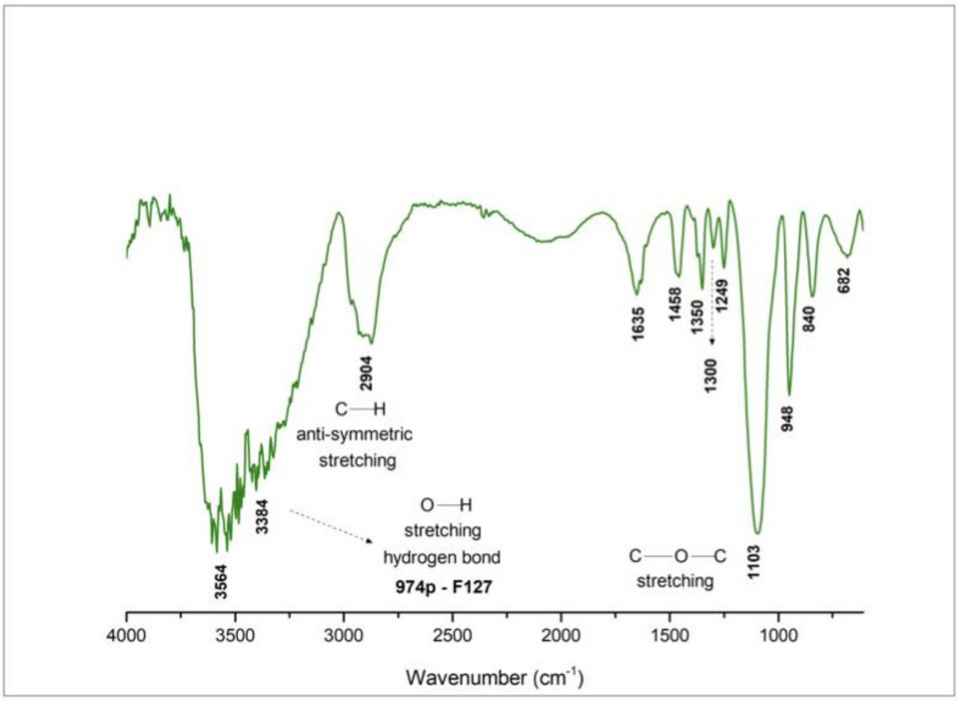

Figure S8. FTIR spectra of empty nanogel nG2002

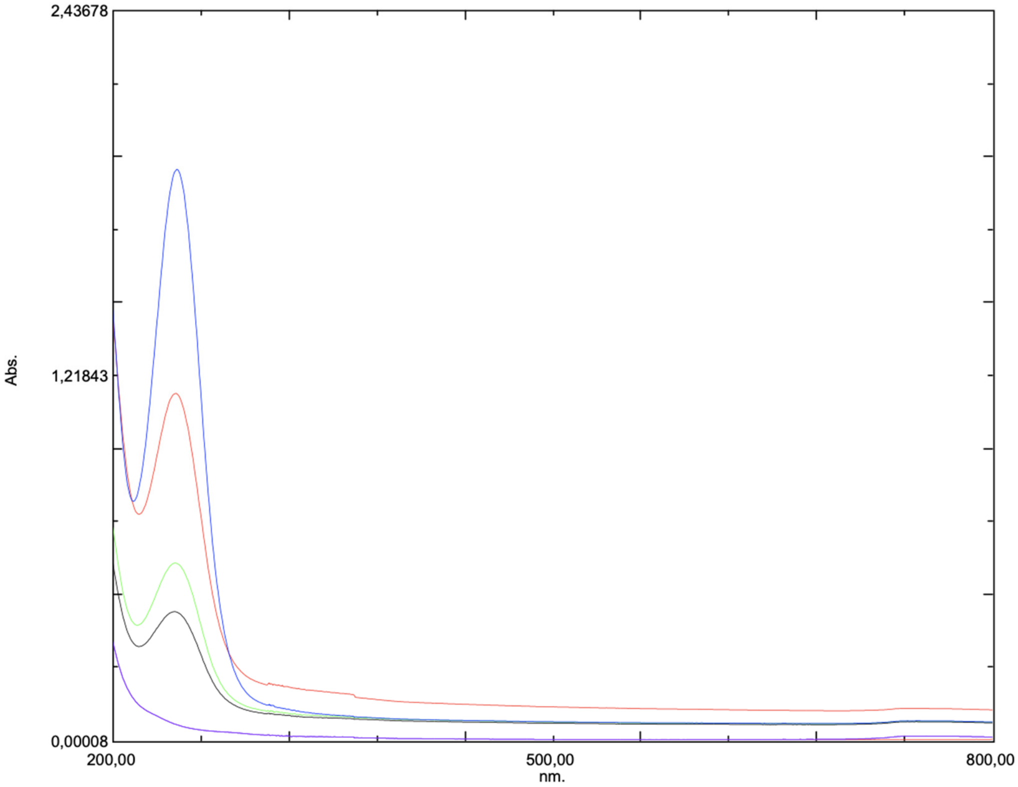

Figure S9. UV−Vis spectrum of nanogel formulations GF1-GF4. The absorbance intensity increases from empty nanogel (GF0 - purple colour curve) to GF4 (nGF2002*Pb* - blue colour curve).

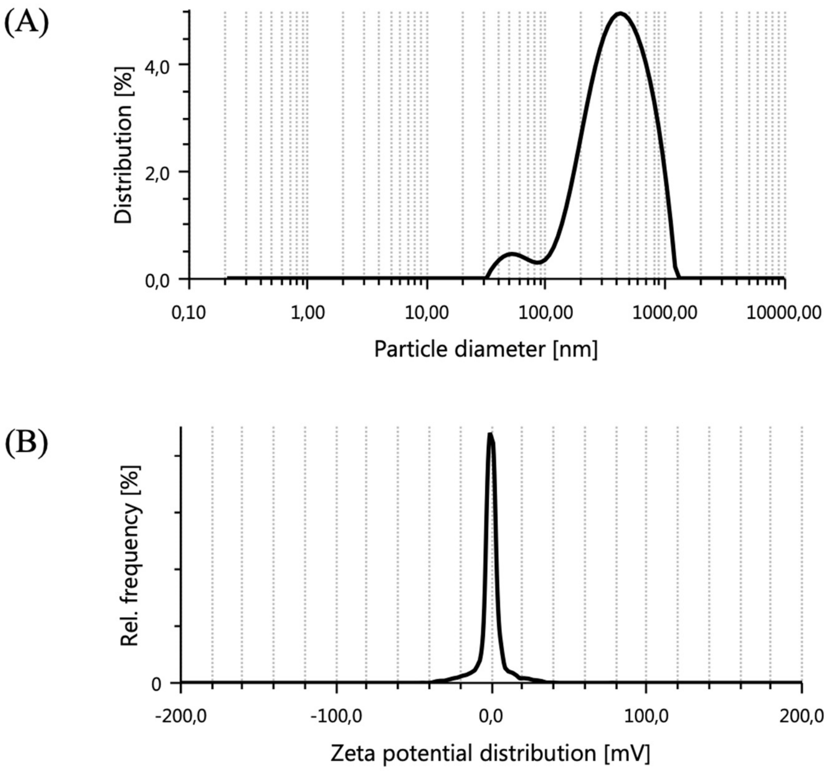

Figure S10. DLS analysis of the nG2002*Pb*. (A) The particle size distribution under low dilution conditions shows a D_H of 429.3 nm and PDI of 0.27. (B) Zeta potential analysis, indicating a value close to zero, suggesting minimal surface charge.
